# Risk of substance-related problems in hypochondriasis

**DOI:** 10.1017/S0033291725103048

**Published:** 2026-01-15

**Authors:** Kayoko Isomura, Susanna Österman, Erik Hedman-Lagerlöf, Ralf Kuja-Halkola, Isabell Brikell, Zheng Chang, Brian M. D’Onofrio, Henrik Larsson, Paul Lichtenstein, David Mataix-Cols, Lorena Fernández de la Cruz, Volen Ivanov, Anna Sidorchuk

**Affiliations:** 1 Center for Psychiatry Research, Department of Clinical Neuroscience, Karolinska Institutet and Stockholm Health Care Services, Region Stockholm, Stockholm, Sweden; 2 Division of Psychology, Department of Clinical Neuroscience, Karolinska Institutet, Stockholm, Sweden; 3 Department of Medical Epidemiology and Biostatistics, Karolinska Institutet, Stockholm, Sweden; 4 Department of Global Public Health and Primary Care, University of Bergen, Bergen, Norway; 5 Department of Biomedicine, Aarhus University, Aarhus, Denmark; 6 Department of Psychological and Brain Sciences, Indiana University, Bloomington, IN, US; 7 School of Medical Sciences, Örebro Universitet, Örebro, Sweden; 8 Department of Clinical Sciences, Lund University, Lund, Sweden

**Keywords:** epidemiology, health anxiety disorder, hypochondriasis, substance-related problems

## Abstract

**Background:**

Hypochondriasis, or health anxiety disorder, is associated with increased mortality, mainly from potentially preventable causes. Substance misuse is a well-known contributor to premature death, yet its relationship with hypochondriasis remains unclear. We assessed the risk of broadly defined substance-related problems in individuals diagnosed with hypochondriasis.

**Methods:**

This Swedish register-based matched cohort study included 4,129 individuals diagnosed with hypochondriasis in specialist services between 1997 and 2020 and 41,290 demographically matched unexposed individuals. Stratified Cox proportional hazards models were fitted to estimate hazard ratios (HRs) for the association between hypochondriasis and substance-related problems – defined as alcohol and drug use disorders, dispensed medications for alcohol dependence and opioid use disorders, and alcohol- and drug-related accidental poisonings, deaths, and suspected criminal offenses. Models were adjusted for sociodemographic variables, parental substance-related problems, and personal psychiatric history.

**Results:**

Substance-related problems were identified in 504 (12.2%) individuals with hypochondriasis and 1,924 (4.7%) matched unexposed individuals. After adjustment for sociodemographic and parental covariates, hypochondriasis was significantly associated with an increased risk of substance-related problems (HR, 2.55; 95% confidence interval [CI], 2.30–2.84). Similar results were observed in individuals without preexisting substance-related problems (HR, 2.85; 95% CI, 2.48–3.27). Further adjustment for psychiatric comorbidity, particularly anxiety and depression, reduced the risk estimates, but the associations remained statistically significant. In an additional analysis including primary care diagnoses of hypochondriasis (presumably reflecting less complex cases), the risk of substance-related problems remained elevated (HR, 1.61; 95% CI, 1.39–1.86).

**Conclusion:**

Improved recognition of, and clinical awareness of substance misuse may help reduce long-term adverse outcomes in individuals with hypochondriasis.

## Introduction

Hypochondriasis, or health anxiety disorder, is characterized by excessive concerns about having or developing a serious illness (World Health Organization, 1992), often accompanied by constant health monitoring, bodily checking, and disease-related internet searches (Scarella, Boland, & Barsky, 2019). Lifetime prevalence ranges from 3–5% in the general population (Sunderland, Newby, & Andrews, 2013; Weck, Richtberg, & Neng, 2014) to 10% in primary care (Tyrer et al., 2013), and up to 20% in specialist settings (Tyrer et al., 2011; Pandey, Parikh, Brahmbhatt, & Vankar, 2017). The disorder is associated with significant distress and impairment and may lead to substantial societal consequences, including increased healthcare utilization and economic burden (Sunderland et al., 2013).

Despite the pervasive fears of illness and death, which are central to hypochondriasis, a recent register-based matched cohort study from Sweden indicated that individuals with hypochondriasis have an increased risk of mortality, mainly from potentially preventable causes (Mataix-Cols et al., 2023). Substance misuse is a well-established contributor to mortality risk, overall and in individuals with psychiatric disorders, including but not limited to schizophrenia, bipolar disorder, and depression (Chesney, Goodwin, & Fazel, 2014; Hjemsæter et al., 2019; Hjorthøj et al., 2015; Roerecke & Rehm, 2013). Although the research on mortality in hypochondriasis is scarce, substance misuse could be assumed to represent one plausible pathway linking hypochondriasis to excess deaths. Several related factors could potentially underlie such an association. For example, hypochondriasis frequently co-occurs with depression and anxiety, both being reported as risk factors for substance misuse and premature mortality (Virtanen et al., 2020; Walker, McGee, & Druss, 2015). Also, the chronic distress associated with health-related anxiety may prompt maladaptive coping with alcohol or drugs (Turner, Mota, Bolton, & Sareen, 2018), and frequent healthcare utilization in individuals with hypochondriasis (Sunderland et al., 2013) may increase their access to and consumption of potentially addictive prescription medications. Better knowledge of disorders and behaviors related to alcohol and drug use in individuals with hypochondriasis is therefore warranted, as such conditions may represent preventable contributors to increased mortality in this patient group.

While comorbidity with depression and anxiety is well-studied for hypochondriasis (Barsky, Wyshak, & Klerman, 1992; Noyes et al., 1994; Scarella et al., 2016), the existing research on the association of hypochondriasis with substance misuse and substance use disorders is limited and inconsistent. For example, a cross-sectional survey-based study on 948 adolescent students showed a higher endorsement of illicit substance use in those who scored above the threshold for ‘hypochondriacal responses’, compared to those who did not (10.1% vs. 5.0%, respectively) (Sirri, Garotti, Grandi, & Tossani, 2015). An online survey study in 758 young adult college students showed that self-reported scores in health anxiety predicted nonmedical use of prescription drugs (Jeffers et al., 2015). Similarly, a cross-sectional study of primary care patients diagnosed with hypochondriasis (*n* = 81) observed that 6.8% had comorbid substance use disorders, although the lack of a control group limits the interpretation of these findings (Fink et al., 2004). By contrast, it has also been hypothesized that hypochondriasis may act as a natural deterrent to substance misuse, as the intense fear of illness might lead individuals to avoid behaviors that pose health risks (Floyd, Prentice-Dunn, & Rogers, 2000; Salkovskis & Warwick, 1986; Schwind, Neng, Höfling, & Weck, 2015). Two case–control studies of primary care patients (*n* = 100 and *n* = 118) found no significant associations between hypochondriasis and substance use disorders (Barsky et al., 1992; Noyes et al., 1994). A comparative cohort study of male psychiatric patients (*n* = 100) reported a lower rate of substance misuse in those with hypochondriasis, compared to patients without hypochondriasis (8% vs. 44%, respectively) (Stenbaeck & Blumenthal, 1964). Similarly, a cohort study of patients with depression (*n* = 402) linked comorbid hypochondriasis with a decreased likelihood of hazardous drinking (Park et al., 2015). These discrepancies in the literature may reflect the studies’ methodological limitations, including small sample sizes, reliance on self-reported data, selection bias, and limited adjustment for psychiatric comorbidities and sociodemographic factors beyond age and sex. It is also worth mentioning that several anxiety disorders (e.g. social anxiety disorder, post-traumatic stress-related disorders, and obsessive-compulsive disorder) and mood disorders (e.g. depression) have been reported to be prospectively associated with an increased risk of subsequent alcohol- and other substance-related problems (Rosenström & Torvik, 2023; Virtanen et al., 2022; Wolitzky-Taylor et al., 2012), while no such prospective studies have been conducted for hypochondriasis.

To address this clear gap in the literature, we conducted the largest nationwide register-based study to date exploring the association between hypochondriasis and risk of substance-related problems. To achieve a more complete characterization of outcome risk and go beyond events contingent on treatment-seeking patterns, we incorporated additional data on alcohol- and drug-related morbidity, mortality, and suspected criminal offenses.

## Methods

The study was approved by the Swedish Ethical Review Authority (reference number 2020–06540). Under Swedish legislation, informed consent is not required for register-based research using pseudonymized data.

### Study design and data sources

In this population-based matched cohort study, we linked nationwide Swedish registers via the unique personal identification numbers assigned to all Swedish residents (Ludvigsson, Otterblad-Olausson, Pettersson, & Ekbom, 2009). Sociodemographic data were extracted from the Census Register (available since 1960), the Total Population Register (since 1968) (Ludvigsson et al., 2016), and the Longitudinal Integration Database for Health Insurance and Labor Market Studies (LISA; since 1990) (Ludvigsson et al., 2019), which together provided information on education, income, civil status, and other demographic variables. Migration data came from the Migration Register, which is part of the Total Population Register, enabling us to account for emigration during follow-up. Clinical data were obtained from the National Patient Register (NPR) (Ludvigsson et al., 2011), which covers inpatient somatic and psychiatric diagnoses since 1969 and 1973, respectively, and all specialist outpatient diagnoses since 2001, and was used to identify hypochondriasis diagnoses, clinical events related to substance-related problems (for study participants and their parents), and psychiatric comorbidities. Diagnoses are coded using the Swedish version of the International Classification of Diseases, eighth (ICD-8; 1969–1986), ninth (ICD-9; 1987–1996), and tenth (ICD-10; 1997 onwards) revisions. Additional hypochondriasis diagnoses were identified from the SRegion Stockholm’s healthcare administration’s database (VAL) (Wändell et al., 2013), covering ICD-10 diagnoses assigned in Stockholm County primary care settings since 1997, thus allowing us to include presumably milder cases managed outside specialist care. Mortality data were extracted from the Cause of Death Register (Brooke et al., 2017), which holds dates and causes of death among Swedish residents since 1952, and was used to ascertain alcohol- and drug-related mortality. Data on prescribed medication use were obtained from the Prescribed Drug Register (Wettermark et al., 2007), where all prescriptions dispensed in pharmacies across Sweden are recorded since July 2005 using Anatomical Therapeutic Chemical (ATC) Classification System codes, and this register was used to identify pharmacological treatment for substance use disorders. Suspected criminal offenses were extracted from the Register of Persons Suspected of Offences (Swedish National Council for Crime Prevention, 2025), which encompasses all reported offenses in Sweden since 1995 in individuals aged 15 years or older (the age of criminal responsibility in Sweden), enabling us to capture substance-related problems outside healthcare. Finally, the biological parents of study participants were identified via the Multi-Generation Register (Ekbom, 2011), which contains kinship data for individuals born since 1932 and residing in Sweden since 1961.

### Study population

The study population included individuals who lived in Sweden at any time between January 1, 1997 (when the ICD-10 was introduced in Sweden) and December 31, 2020 (end of the study). We excluded individuals who died or emigrated before 1997 or their 10th birthday (minimal age for clinical substance-related outcomes) (Virtanen et al., 2021), whichever occurred last, or if they were born after December 31, 2010 (i.e. <10 years before the study end).

Individuals were considered exposed if they had at least one hypochondriasis diagnosis recorded in the NPR between 1997 and 2020. The date of the first diagnosis denoted the index date. Each exposed individual was randomly matched with 10 unexposed individuals from the study population who had no records of hypochondriasis by the date when the exposed individual received the diagnosis. Unexposed individuals were assigned the same index date as their exposed counterparts. Matching was done on sex, birth year, and county of residence at the index date.

For the additional analysis, the study cohort was restricted to individuals residing in Stockholm County between 1997 and 2020 at the age of 10 years or older. In this sub-cohort, the exposed group consisted of individuals with hypochondriasis diagnosed in either specialist services (recorded in the NPR) or primary care (recorded in the VAL). Each exposed person was matched with up to 10 unexposed Stockholm County residents using the same matching criteria as above.

In the main and additional analyses, the exposed and matched unexposed individuals were followed up from the index date or their 10th birthday, whichever occurred last, until the date of outcome, death from other causes, emigration, or December 31, 2020, whichever came first. For unexposed individuals, follow-up was additionally censored at the date they changed exposure status (if they received a hypochondriasis diagnosis during follow-up).

### Exposure

Individuals with hypochondriasis (Swedish ICD-10 code F45.2) were identified from the NPR if diagnosed in inpatient or specialist outpatient care at the age of 6 years or older (to avoid diagnostic misclassification). The ICD-10 code for hypochondriasis in the NPR has been validated and deemed acceptable for register-based research (Rautio et al., 2021). To further minimize the risk of misdiagnosis, we excluded individuals diagnosed with dysmorphophobia (Swedish ICD-10 code F45.2A) from the entire study cohort due to the proximity of the diagnostic codes (Rautio et al., 2021).

In the sub-cohort of Stockholm County residents, we attempted to improve the coverage of hypochondriasis by including primary care diagnoses from the VAL in addition to the records of inpatient and specialist outpatient diagnoses of hypochondriasis from the NPR. Primary care hypochondriasis diagnoses were collected if recorded between January 1, 1997, and December 31, 2020, at the age of 6 years or older. Primary care data were not available for other Swedish regions, which led to restricting the sub-cohort to Stockholm County residents.

### Outcomes

We collected data on substance-related problems recorded during the follow-up, using clinical, pharmacological, and behavioral (i.e. suspected offenses) measures (Quinn et al., 2020). The outcomes were defined as a combined variable ‘any substance-related problem’ (if any alcohol- and/or drug-related problems were recorded), and, in addition, as ‘alcohol-related problems’ and ‘drug-related problems’ separately to assess whether the association with hypochondriasis differed by substance type. Specifically, we retrieved data from the NPR using ICD-10 codes for alcohol and drug use disorders and accidental poisoning, recorded in inpatient or specialist outpatient care (as main or secondary diagnoses). The same ICD-10 codes were used in the Cause of Death Register to identify alcohol- and drug-related deaths (as underlying or contributing causes). Furthermore, from the Prescribed Drug Register, we retrieved dispensation records with the ATC codes for medications used for the treatment of alcohol dependence and opioid use disorders. To avoid misdiagnosis, clinical and pharmacological data were retrieved if recorded at the age of 10 years or older. Finally, we collected data from the Register of Persons Suspected of Offenses on suspected alcohol- and drug-related criminal offenses recorded at the age of 15 years or older. Supplementary Table S1 details the register data used to define the outcomes.

### Covariates

*A priori* selection of covariates for statistical modeling was based on factors related to hypochondriasis (or proxies for such factors due to the scarcity of research on specific risk factors for hypochondriasis) (Kikas, Werner-Seidler, Upton, & Newby, 2024; Rachman, 2012; Thorgaard, Frostholm, & Rask, 2018) and factors associated with substance-related problems (Carvalho et al., 2019; Lin et al., 2024) that fulfilled the criteria for confounding (Greenland & Lash, 2008). Specifically, from the Total Population Register, we extracted place of birth (Sweden vs. abroad). From the Census and LISA registers, we gathered data on the highest attained education level (elementary [≤9 years], secondary [10–12 years], and higher education [>12 years]), civil status (single/divorced/widowed and married/cohabiting), and disposable household income (lowest 20%, middle 60%, and top 20%), for which we used the latest record available before the index date. From the NPR, we collected data on psychiatric disorders, if diagnosed before or on the index date, grouped as (1) neurodevelopmental disorders, (2) psychotic disorders, (3) bipolar disorders, (4) depressive disorders, (5) anxiety-related disorders, and (6) eating disorders (Supplementary Table S2). Using the Multi-Generation Register, we linked participants to their biological parents and then gathered maternal and paternal lifetime records of substance-related problems, which are known to be associated with offspring anxiety-related disorders and substance use disorders (Zhou et al., 2024), using the same definition and registers as for the outcomes. Finally, we collected data on the individuals’ preexisting substance-related problems recorded before the index date, using the same definitions as for the outcomes.

### Statistical analysis

We fitted stratified Cox proportional hazards regression models to estimate hazard ratios (HRs) and 95% confidence intervals (CIs) for the association between hypochondriasis and any substance-related problems and, separately, alcohol- and drug-related problems. Time since follow-up start was the underlying timescale. Missing data in socioeconomic and parental covariates were coded as unknown and included in the models as nominal variables.

The main analysis focused on hypochondriasis diagnoses from specialist services recorded in the NPR. First, all cohort members, regardless of preexisting substance-related problems, were analyzed. We fitted a minimally adjusted model (Model 1), controlling for the matching variables (i.e. sex, birth year, and county of residence). This was followed by a model additionally adjusted for sociodemographic covariates, including country of birth, education, civil status, and income (Model 2), and by a model with additional adjustment for parental substance-related problems (Model 3). The analyses were also conducted separately for males and females. Second, the models were re-ran in a sub-cohort restricted to individuals without preexisting substance-related problems to ensure that the outcomes reflected incident cases. Third, we explored the influence of psychiatric disorder history by re-running Model 2 with additional adjustment for psychiatric disorders (one disorder group at a time). Model 2 was chosen over Model 3 to avoid the impact of missing data on parental substance-related problems.

In an additional analysis, we repeated the main analyses in the sub-cohort of Stockholm County residents, which enabled the inclusion of primary care diagnoses of hypochondriasis. Because the number of individuals diagnosed with hypochondriasis exclusively in primary care was relatively small, we constructed the exposure variable by combining primary care and specialist service-diagnosed hypochondriasis to increase the statistical power of the analysis and ensure the reliability of the estimates.

Two sensitivity analyses were performed. First, in line with prior literature (Abrahamsson, Berge, Öjehagen, & Håkansson, 2017; Quinn et al., 2020), we re-ran Models 1–3 in the whole cohort using a stricter outcome definition by excluding dispensations for opioid use disorder treatment to avoid misclassification with medication for pain relief. Second, Models 1–3 were re-run after restricting the study cohort to individuals with no missing data on any covariates.

Data management and analyses were performed using SAS, version 9.4 (SAS Institute Inc., Cary, NC). All tests used a two-tailed significance set at *P* < 0.05.

## Results

### Cohort description

**Figure 1.**
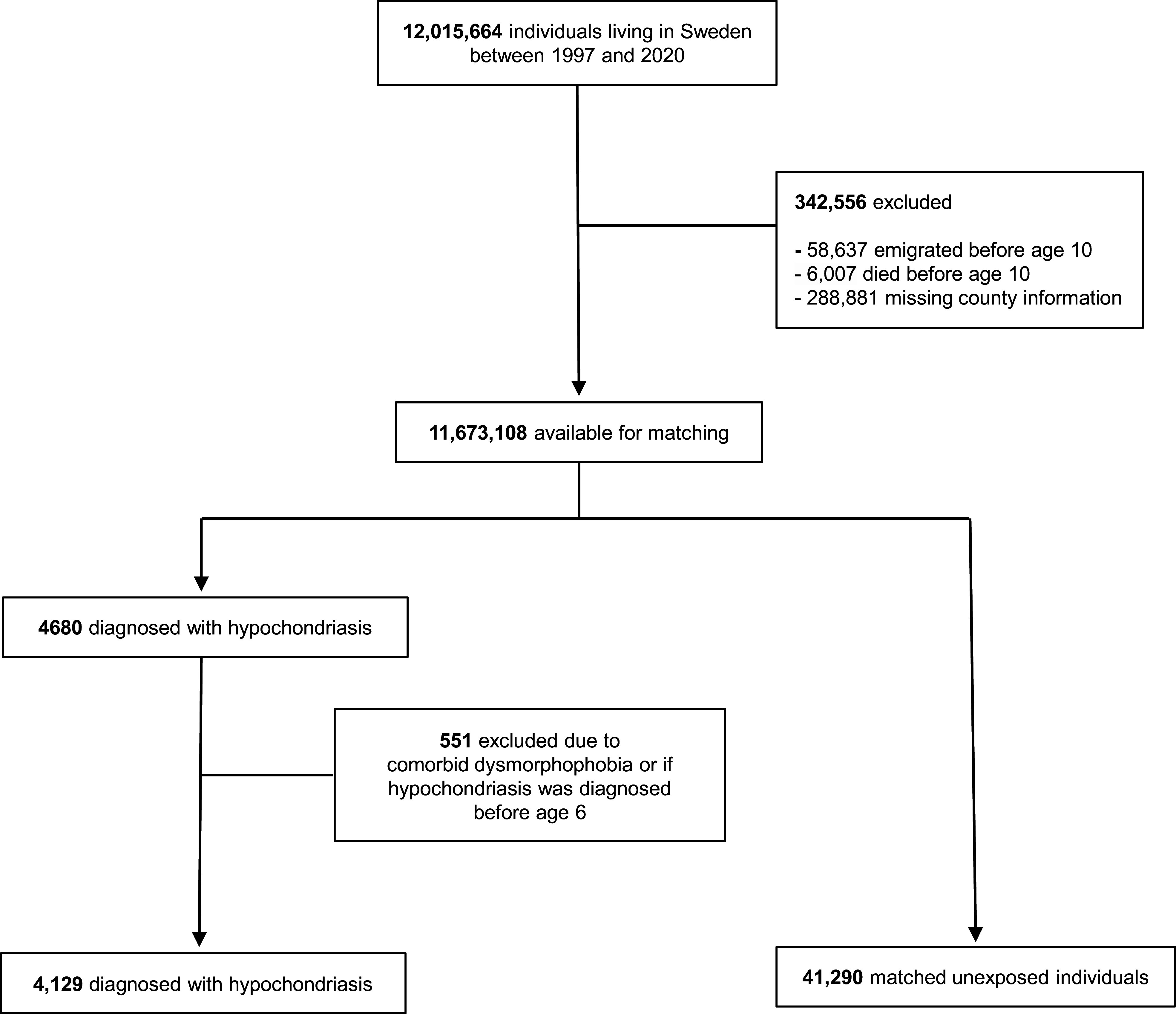
Selection of individuals with a diagnosis of hypochondriasis and matched unaffected individuals.

Out of 12,015,664 individuals living in Sweden between 1997 and 2020, 11,673,108 were available for matching after excluding those who died or emigrated before age 10 years or had missing data on county of residence (Figure 1). Among them, 4,680 persons had a hypochondriasis diagnosis in specialist services and recorded in the NPR in 1997–2020, but 551 were further excluded due to receiving the diagnosis before age 6 years or also having a record of dysmorphophobia. In total, 4,129 individuals with hypochondriasis were available for analyses and were matched to 41,290 unexposed individuals (43.3% female; mean age at first diagnosis among the exposed 37.7 years, standard deviation [SD], 15.4). Among individuals diagnosed with hypochondriasis in specialist services, 3,468 (84.0%) received their diagnosis in psychiatric units, whereas 661 (16.0%) were diagnosed in somatic specialist care units. Table 1 presents the cohort characteristics. Compared to unexposed counterparts, individuals with hypochondriasis were more likely to be Swedish-born, unmarried/not cohabiting, have a lower level of education and income, and have higher rates of parental substance-related problems, own preexisting substance-related problems, and a history of psychiatric disorders.

**Table 1.** Distribution of sociodemographic, clinical, and parental characteristics of the study participants

	Individuals with hypochondriasis *n* = 4,129^a^	Matched unexposed individuals *n* = 41,290	*χ*^2^ test or *t*-test	*p*-value
	*n* (%)	*n* (%)		
**Sex**			NA	NA
Female	2,342 (56.72)	23,420 (56.72)		
Male	1,787 (43.28)	17,870 (43.28)		
**Years of birth**			NA	NA
≤1939	182 (4.41)	1,820 (4.41)		
1940–1959	573 (13.88)	5,730 (13.88)		
1960–1979	1,429 (34.61)	14,290 (34.61)		
1980–1999	1,846 (44.71)	18,460 (44.71)		
≥2000	99 (2.40)	990 (2.40)		
**Age at the index date^b^, years,** **mean (SD)^c^**			NA	NA
Among all individuals	37.68 (15.41)	37.68 (15.41)		
Among females	37.81 (15.45)	37.81 (15.45)		
Among males	37.52 (15.36)	37.52 (15.36)		
**Place of birth**			130.54	<0.0001
Sweden	3,568 (86.41)	32,576 (78.90)		
Abroad	561 (13.59)	8,714 (21.10)		
**Educational level**			37.94	<0.0001
Elementary education	923 (22.35)	8,176 (19.80)		
Secondary education	1,595 (38.63)	16,980 (41.12)		
Higher education	1,576 (38.17)	15,399 (37.29)		
Unknown/missing	35 (0.85)	735 (1.78)		
**Civil status**			76.03	<.0001
Single, divorced, or widowed	2,992 (72.46)	27,151 (65.76)		
Married or cohabiting	1,125 (27.25)	14,018 (33.95)		
Unknown/missing	12 (0.29)	121 (0.29)		
**Disposable household income level**			196.45	<0.0001
Lowest 20%	1,217 (29.47)	8,418 (20.39)		
Middle 60%	2,224 (53.86)	24,462 (59.24)		
Top 20%	666 (16.13)	7,938 (19.22)		
Unknown/missing	22 (0.53)	472 (1.14)		
**Maternal substance-related problems**			163.4398	<.0001
No	3,414 (82.68)	31,950 (77.38)		
Yes	259 (6.27)	1,746 (4.23)		
Unknown/missing	456 (11.04)	7,594 (18.39)		
**Paternal substance-related** **problems**			116.8396	<.0001
No	3,078 (74.55)	29,123 (70.53)		
Yes	479 (11.60)	3,733 (9.04)		
Unknown/missing	572 (13.85)	8,434 (20.43)		
**Preexisting alcohol-related problems**			87.77	<0.0001
No	3,823 (92.59)	39,542 (95.77)		
Yes	306 (7.41)	1,748 (4.23)		
**Preexisting drug-related problems**			188.85	<0.0001
No	3,796 (91.94)	39,785 (96.36)		
Yes	333 (8.06)	1,505 (3.64)		
**History of psychiatric disorders**				
Any psychiatric disorders	2,832 (68.59)	4,134 (10.01)	9918.64	<0.0001
Neurodevelopmental disorders	400 (9.69)	959 (2.32)	701.47	<0.0001
Psychotic disorders	224 (5.43)	339 (0.82)	646.94	<0.0001
Bipolar disorders	209 (5.06)	364 (0.88)	526.55	<0.0001
Depressive disorders	1,475 (35.72)	2,149 (5.20)	4761.40	<0.0001
Anxiety-related disorders	2,144 (51.93)	1,976 (4.79)	10112.65	<0.0001
Eating disorders	247 (5.98)	467 (1.13)	570.88	<0.0001
**Age at the first recorded substance-related problem, years, mean (SD)**				
Among all individuals	39.32 (15.58)	37.46 (14.98)	−2.45	0.0142
Among females	40.46 (16.76)	38.66 (15.28)	−1.47	0.141
Among males	38.46 (14.61)	36.81 (14.78)	−1.71	0.0868
**Duration of follow-up, years, mean (SD)**				
For all individuals	6.77 (5.47)	7.47 (5.66)	7.66	<0.0001
For females	6.89 (5.51)	7.49 (5.72)	4.88	<0.0001
For males	6.61 (5.43)	7.45 (5.59)	6.08	<0.0001

*Note:* Statistical comparisons were conducted using Chi-squared tests for categorical variables and *t*-tests for continuous variables; *p*-values from the corresponding tests are provided in the last column. Chi-squared/*t*-tests and *p*-values that correspond to variables used for matching are reported as ‘NA’ – not applicable.

a Among 4,129 individuals diagnosed with hypochondriasis in specialist services, 3,468 (84.0%) received their diagnosis in psychiatric units, whereas 661 (16.0%) were diagnosed in somatic specialist care.

b Index date refers to the date of the first diagnosis of hypochondriasis among the exposed individuals and the corresponding date among their matched unexposed counterparts.

c Age at the index date is reported for illustrative purposes, while year of birth was used as a variable for matching.

Abbreviations: SD, standard deviation; NA, not applicable.

### Main cohort analyses

Among 4,129 individuals with hypochondriasis in the full cohort, 504 (12.2%) had a record of any substance-related problem during follow-up, compared to 1,924 (4.7%) among 41,290 unexposed individuals (mean follow-up of 6.8 years [SD, 5.5] and 7.5 years [SD, 5.7], respectively). This corresponded to crude incidence rates (IRs) of 18.0 and 6.2 per 1,000 person-years, respectively. The minimally adjusted model showed a statistically significant association between hypochondriasis and substance-related problems, with nearly a threefold increased risk (HR, 2.89; 95% CI, 2.62–3.19), which remained after adjusting for sociodemographic factors (Model 2: HR, 2.59; 95% CI, 2.33–2.87) and parental substance-related problems (Model 3: HR, 2.55; 95% CI, 2.30–2.84) (Table 2). Separate analyses for alcohol- and drug-related problems revealed associations of similar magnitude (e.g. Model 3: HRs, 2.43; 95% CI, 2.12–2.78 and 2.81; 95% CI, 2.46–3.22, respectively).

**Table 2.** Hazard ratios (HRs) with 95% confidence intervals (CIs) for any substance-related problems and alcohol- and drug-related problems among individuals with hypochondriasis, compared to matched unexposed individuals

	Individuals with hypochondriasis	Matched unexposed individuals	Model 1^a^	Model 2^b^	Model 3^c^
	*n* (%)	*n* (%)	HR (95% CI)	HR (95% CI)	HR (95% CI)
**Whole cohort**					
Included individuals	4,129 (100)	41,290 (100)			
Any substance-related problem	504 (12.21)	1,924 (4.66)	2.89 (2.62–3.19)	2.59 (2.33–2.87)	2.55 (2.30–2.84)
Alcohol-related problem	294 (7.12)	1,140 (2.76)	2.76 (2.42–3.14)	2.46 (2.15–2.81)	2.43 (2.12–2.78)
Drug-related problem	328 (7.94)	1,100 (2.66)	3.21 (2.83–3.63)	2.88 (2.52–3.29)	2.81 (2.46–3.22)
**Without preexisting substance-related problems^d^**					
Included individuals	3,612 (100)	38,540 (100)			
Any substance-related problem	288 (7.97)	1,112 (2.89)	3.06 (2.67–3.50)	2.88 (2.51–3.30)	2.85 (2.48–3.27)
Alcohol-related problem	153 (4.24)	682 (1.77)	2.59 (2.16–3.09)	2.42 (2.02–2.91)	2.40 (2.00–2.89)
Drug-related problem	176 (4.87)	553 (1.43)	3.60 (3.02–4.29)	3.38 (2.82–4.05)	3.31 (2.75–3.98)

a Model 1 is adjusted for the matching variables (sex, birth year, and county of residence at the index date).

b Model 2 is additionally adjusted for sociodemographic factors (place of birth, level of education, civil status, and household income).

c Model 3 is additionally adjusted for maternal and paternal substance-related problems.

d Restricted to individuals with neither preexisting alcohol- nor drug-related problems.

In individuals without preexisting substance-related problems, incident substance-related outcomes were observed in 288 (8.0%) out of 3,612 exposed individuals and in 1,112 (2.9%) out of 38,540 unexposed individuals (crude IR: 11.2 vs. 3.8 per 1,000 person-years). The corresponding HRs were similar to those in the full cohort (Table 2).

In sex-stratified analyses, the HRs for the association of hypochondriasis with any substance-related problems and alcohol-related problems were similar in females and males (overlapping 95% CIs in all models, except in Model 1 for any substance-related problems). The associations with drug-related problems were stronger in females than in males in all models (nonoverlapping 95% CIs) (Supplementary Table S3).

Adjustments for psychiatric history attenuated the risk of all outcomes in both the full cohort and in the cohort of individuals without preexisting substance-related problems (Table 3). The largest attenuation occurred after adjusting for a history of depressive and anxiety disorders, but all HRs remained statistically significant.

**Table 3.** Hazard ratios (HRs) with 95% confidence intervals (CIs) for any substance-related problems, alcohol- and drug-related problems among individuals with hypochondriasis, compared to matched unexposed individuals, further adjusted for different groups of psychiatric comorbidities

	Initial Model 2^a^ additionally adjusted for the following disorder groups (one group at a time)					
	Neurodevelopmental disorders	Psychotic disorders	Bipolar disorders	Depressive disorders	Anxiety disorders	Eating disorders
	HR (95% CI)	HR (95% CI)	HR (95% CI)	HR (95% CI)	HR (95% CI)	HR (95% CI)
**Whole cohort**						
Any substance-related problem	2.39 (2.15–2.66)	2.45 (2.20–2.72)	2.44 (2.19–2.71)	1.79 (1.59–2.02)	1.66 (1.46–1.89)	2.52 (2.27–2.80)
Alcohol-related problem	2.21 (1.92–2.54)	2.36 (2.06–2.71)	2.32 (2.02–2.66)	1.79 (1.54–2.08)	1.63 (1.38–1.92)	2.40 (2.10–2.76)
Drug-related problem	2.64 (2.30–3.02)	2.68 (2.34–3.08)	2.69 (2.34–3.08)	1.92 (1.65–2.23)	1.80 (1.53–2.12)	2.79 (2.43–3.20)
**Without preexisting substance-related problems^b^**						
Any substance-related problem	2.82 (2.46–3.24)	2.81 (2.45–3.24)	2.73 (2.37–3.14)	2.28 (1.95–2.66)	2.32 (1.96–2.74)	2.81 (2.45–3.23)
Alcohol-related problem	2.35 (1.95–2.83)	2.46 (2.04–2.96)	2.35 (1.95–2.83)	2.11 (1.72–2.59)	2.09 (1.67–2.62)	2.37 (1.97–2.85)
Drug-related problem	3.32 (2.75–3.99)	3.20 (2.66–3.85)	3.12 (2.59–3.76)	2.42 (1.97–2.98)	2.61 (2.10–3.26)	3.30 (2.74–3.97)

a Model 2 is adjusted for the matching variables (sex, birth year, and county of residence at the index date) and sociodemographic factors (place of birth, level of education, civil status, and household income).

b Restricted to individuals with neither preexisting alcohol- nor drug-related problems.

### Stockholm County sub-cohort analyses

Among 2,946,706 individuals who lived in Stockholm County in 1997–2020 and were eligible for inclusion in the Stockholm County sub-cohort, 3,343 individuals had a record of hypochondriasis (diagnosed in specialist or primary care). The majority of these individuals were diagnosed exclusively in specialist services (*n* = 1,753; 52.4%), followed by those diagnosed only in primary care (*n* = 1,235; 36.9%), and by a smaller group who were diagnosed in both settings (*n* = 355; 10.6%) (Supplementary Table S4). When 3,343 exposed individuals were matched to 33,419 unexposed individuals, substance-related problems were identified in 233 (7.0%) and 1,361 (4.1%) members of these groups, respectively (crude IR: 14.8 vs. 8.4 per 1,000 person-years). The HRs estimated in the sub-cohort were attenuated, compared to the main analyses in the full cohort, but remained significant across all outcomes (e.g. Model 3 for any substance-related problem: HR, 1.61; 95% CI, 1.39–1.86) (Table 4). When the sub-cohort was further restricted to individuals without preexisting substance-related problems, the outcomes were observed in 113 (3.8%) out of 2,983 individuals with hypochondriasis and in 689 (2.3%) out of 30,564 unexposed individuals (crude IR: 7.8 versus 4.5 per 1,000 person-years). All HRs were similar to those before the restriction (Table 4). Adjustment for depression and anxiety disorders attenuated the associations to the null, while HRs remained significant after controlling for all other psychiatric disorders (Supplementary Table S5).

**Table 4.** Hazard ratios (HRs) with 95% confidence intervals (CIs) for any substance-related problems and alcohol- and drug-related problems, among individuals from the sub-cohort of Stockholm County residents with a diagnosis of hypochondriasis assigned in specialist services or in primary care, compared to matched unexposed individuals

	Individuals with hypochondriasis	Matched unexposed individuals	Model 1^a^	Model 2^b^	Model 3^c^
	*n* (%)	*n* (%)	HR (95% CI)	HR (95% CI)	HR (95% CI)
**Sub-cohort of individuals residing in Stockholm County in 1997–2020**					
Included individuals	3,343 (100)	33,419 (100)			
Any substance-related problem	233 (6.97)	1361 (4.07)	1.75 (1.52–2.01)	1.63 (1.41–1.88)	1.61 (1.39–1.86)
Alcohol-related problem	145 (4.34)	789 (2.36)	1.85 (1.55–2.21)	1.69 (1.41–2.03)	1.67 (1.39–2.01)
Drug-related problem	137 (4.10)	767 (2.30)	1.79 (1.49–2.15)	1.74 (1.43–2.11)	1.70 (1.40–2.07)
**Without preexisting substance-related problems** ^d^					
Included individuals	2,983 (100)	30,564 (100)			
Any substance-related problem	113 (3.79)	689 (2.25)	1.74 (1.42–2.13)	1.67 (1.36–2.05)	1.65 (1.34–2.02)
Alcohol-related problem	64 (2.15)	419 (1.37)	1.60 (1.22–2.08)	1.51 (1.16–1.98)	1.47 (1.12–1.94)
Drug-related problem	66 (2.21)	331 (1.08)	2.10 (1.61–2.75)	2.04 (1.55–2.69)	2.05 (1.55–2.72)

a Model 1 is adjusted for the matching variables (sex, birth year, and county of residence at the index date).

b Model 2 is additionally adjusted for sociodemographic factors (place of birth, level of education, civil status, and household income).

c Model 3 is additionally adjusted for maternal and paternal substance-related problems.

d Restricted to individuals from the sub-cohort of Stockholm County residents with neither preexisting alcohol- nor drug-related problems.

### Sensitivity analyses

Excluding dispensation records of opioid use disorder medications from the outcome definition, as well as restricting the analyses to individuals with complete data on all covariates, did not significantly alter the results (Supplementary Table S6).

## Discussion

In this nationwide matched cohort study, we found a statistically significant association between hypochondriasis and broadly defined substance-related problems. Individuals diagnosed with hypochondriasis in specialist services had more than a 2.5-fold increased outcome risk, compared to matched unexposed individuals, even after controlling for sociodemographic factors and parental substance-related problems. Elevated risks persisted when alcohol- and drug-related outcomes were assessed separately, and in the analyses of individuals without preexisting substance-related problems. Adjustment for history of psychiatric comorbidity, particularly depression and anxiety, attenuated but did not eliminate the associations of interest. We also observed similar risk estimates among males and females, except for drug-related problems, where the association with hypochondriasis was stronger in females, possibly due to the low absolute risk in unexposed females. Finally, when primary care diagnoses of hypochondriasis (likely representing milder or less complex cases) were added and analyzed in a sub-cohort of Stockholm County residents, risk estimates were attenuated but remained statistically significant across all models. The only exception was the additional adjustment for depression and anxiety disorders, which resulted in statistically nonsignificant HRs. However, it is worth mentioning that when Stockholm County sub-cohort members without preexisting substance-related problems were analyzed, the increased risk of any substance-related problems and drug-related problems persisted even when controlling for a history of these comorbidities.

Direct comparisons with previous studies are difficult due to the scarcity and inconsistency of previous evidence. Earlier research even suggested that hypochondriasis might be associated with a lower risk of substance misuse (Barsky et al., 1992; Floyd et al., 2000; Noyes et al., 1994; Park et al., 2015; Salkovskis & Warwick, 1986; Schwind et al., 2015; Stenbaeck & Blumenthal, 1964), since health concerns could deter individuals from engaging in risky behaviors. Our results challenge that idea. Our findings may help explain the previously observed increase in mortality from natural and external causes among individuals with hypochondriasis (Mataix-Cols et al., 2023). Alcohol and drug use may contribute to both suicide risk (Arsenault-Lapierre, Kim, & Turecki, 2004; Brady, 2006; Pitman, Krysinska, Osborn, & King, 2012) and natural causes of death (e.g. due to cardiovascular disease) (Krittanawong et al., 2022; Roerecke & Rehm, 2014).

More broadly, our findings are in line with evidence linking psychiatric disorders in general to substance-related outcomes. For example, both anxiety- and mood-related disorders are associated with an increased risk of subsequent alcohol and drug misuse (Goldfield, Zhang, & George, 2024; Lai, Cleary, Sitharthan, & Hunt, 2015; Virtanen et al., 2020). Possible explanations for the associations observed in our study could reflect that individuals with hypochondriasis may use alcohol and drugs as maladaptive coping strategies to alleviate anxiety (Andrade et al., 2014; Khantzian, 1997). Similar assumptions on substance misuse as possible coping strategies were also made in studies on other psychiatric disorders (Goldfield et al., 2024; Grant et al., 2016; Kessler, 2004; Lai et al., 2015; Smith & Book, 2008; Virtanen et al., 2020; Virtanen et al., 2022). Another explanation may relate to shared familial vulnerabilities to both hypochondriasis and substance misuse (Castillo-Carniglia, Keyes, Hasin, & Cerdá, 2019; Fabricius, Langa, & Wilson, 2008), and the fact that patients may experience a mutual reinforcement between anxiety symptoms and substance use over time (Fabricius et al., 2008; Turner et al., 2018). In addition, frequent healthcare seeking (Sunderland et al., 2013) could increase access to prescription drugs, raising the risk of misuse (Hasin & Katz, 2007). These potential explanations warrant further investigation.

From a clinical perspective, our findings highlight the importance of identifying and addressing substance-related problems in individuals with hypochondriasis. Since substance misuse may partly reflect anxiety-related coping, early treatment of hypochondriasis could help reduce these risks (Brown et al., 1997). In parallel, providing timely support and integrated treatment approaches that target both hypochondriasis and substance misuse may help improve long-term outcomes (Kelly & Daley, 2013). Importantly, current clinical guidelines, such as those from the US Preventive Services Task Force, recommend that healthcare providers screen all adult patients in primary care settings for unhealthy alcohol use, as early detection and brief interventions can reduce substance-related harms (U.S. Preventive Services Task Force, 2018). Our findings underscore the importance of adhering to these recommendations and ensuring that individuals with hypochondriasis are not overlooked. Considering the relatively high IRs of substance-related problems and substantially elevated risks in individuals with hypochondriasis, early identification of and intervention toward preventing substance misuse in these patients should be prioritized.

### Strengths and limitations

Strengths of this study include its large population-based sample, the use of clinically validated diagnoses of hypochondriasis (Rautio et al., 2021), and reliance on nationwide registers with prospectively collected health and administrative data for gathering information on study variables. Altogether, these approaches minimized selection, recall, and reporting biases and allowed for rigorous control of a broad range of potential confounders.

Several limitations should be considered. First, the register data on hypochondriasis only capture individuals who sought medical treatment, potentially limiting the generalizability of our findings. The number of individuals diagnosed with hypochondriasis in our study was considerably lower than expected based on international population-based prevalence estimates of 3–5% (Sunderland et al., 2013; Weck et al., 2014). This might indicate that hypochondriasis is underdiagnosed in Sweden or reflect differences in diagnostic and coding practices between countries and variations in register coverage across healthcare settings and calendar years (e.g. the NPR covers outpatient specialist diagnoses from 2001 and does not include primary care diagnoses). In turn, this may have resulted in some individuals with hypochondriasis being misclassified as unexposed. However, while the diagnoses of hypochondriasis recorded in the NPR may reflect more severely ill individuals with higher psychiatric comorbidity, our previous validation study (Rautio et al., 2021) indicated a reasonable distribution of symptom severity among those diagnosed in specialist services. Moreover, we attempted to address this potential limitation by also including hypochondriasis diagnoses from primary care, and the results of this additional analysis supported the main findings. Second, frequent healthcare contacts among individuals with hypochondriasis may have led to a higher likelihood of detecting substance-related morbidity, contributing to possible surveillance bias. To mitigate this, we included outcomes less dependent on healthcare utilization (e.g. substance-related death and criminal offenses). Third, missing data on socioeconomic and parental variables could have affected the estimates, but this was addressed in a sensitivity analysis of individuals with complete covariate data, which yielded identical results. Finally, the end of follow-up overlapped with the beginning of the coronavirus disease 2019 pandemic in Sweden, which may have potentially influenced mental health, substance use, and healthcare utilization. However, several Swedish population-based studies reported a relative stability in rates of mental healthcare service utilization during the early pandemic period and specialist services-diagnosed substance use dependencies compared to those seen in Sweden in pre-pandemic years (Kisiel et al., 2025; Lieber & Werneke, 2025). Also, the pandemic period represented <2 years of our 24-year long follow-up, making it unlikely that our results were substantially influenced by this brief overlap.

## Conclusions

Individuals with hypochondriasis have a substantially increased risk of substance-related problems compared to unexposed individuals, and this association is not fully explained by socioeconomic covariates, previous history of other psychiatric disorders, or parental substance-related problems. Our findings underscore the need for clinical vigilance regarding alcohol and drug use in this patient group. Recognizing and addressing substance misuse may be an important step toward improving prognosis and reducing preventable mortality in individuals with hypochondriasis.

## Supporting information

10.1017/S0033291725103048.sm001 Isomura et al. supplementary material Isomura et al. supplementary material

## Data Availability

The Public Access to Information and Secrecy Act in Sweden prohibits the authors from making individual-level data publicly available. Researchers who are interested in replicating our work can apply for de-identified individual-level data from the register holders.

## Supplementary material

The supplementary material for this article can be found at http://doi.org/10.1017/S0033291725103048.
