# Supplementary material for: Risk of substance-related problems in hypochondriasis
Source: Psychol Med. 2026 Jan 15;56:e22. doi: 10.1017/S0033291725103048 (PMC12885346; doi:10.1017/S0033291725103048)
Supplement: Isomura et al. supplementary material [file S0033291725103048sup001.docx]

**Supplementary Table S1**. ICD-codes, ATC-codes, and criminal offences codes for the ascertainment of substance-related outcomes, including alcohol- and drug-related problems^a^

| **Any alcohol-related problems** | |
| --- | --- |
| Alcohol use disorders and poisoning by alcohol (from the Nation Patient Register) and deaths due to alcohol use disorders or poisoning by alcohol (from the Cause of Death Register)^b^ | *ICD-10 codes:*  F10 (Mental and behavioural disorders due to use of alcohol)  T51.0 (Toxic effect of ethanol),  X45 (Accidental poisoning by and exposure to alcohol)  *ICD-9 codes:*  291 (Alcoholic psychosis),  303 (Alcoholism/ dependence),  305A (Alcohol misuse)  980A (Toxic effect of ethanol)  *ICD-8 codes:*  291 (Alcoholic psychosis),  303 (Alcoholism/ dependence)  980,00 (Toxic effect of ethanol),  980,01 (Toxic effect of ethanol surrogates) |
| Medications used for alcohol dependence treatment^c^ (from the Prescribed Drug Register) | *ATC-codes:*  N07BB01 (disulfiram), N07BB03 (acamprosate), N07BB04 (naltrexone), N07BB05 (nalmefene) |
| Suspected criminal offences related to alcohol use (from the Register of Persons Suspected of Offences) | *Codes for suspected criminal offences:*  3005 (Driving under the influence of only alcohol, or both alcohol and drugs; *from the Law 1951:649, paragraphs 4 and 4a*),  3201 (Operating maritime vessel under the influence of alcohol or other drugs; *from the Law 1994:1009, chapter 20, paragraphs 4 and 5*) |
| **Any drug-related problems** | |
| Drug use disorders and poisoning by drugs (from the Nation Patient Register) and deaths due to drug use disorders or poisoning by drugs (from the Cause of Death Register)^b^ | *ICD-10 codes:*  F11 (Mental and behavioural disorders due to use of opioids)  F12 (Mental and behavioural disorders due to use of cannabinoids),  F13 (Mental and behavioural disorders due to use of sedatives or hypnotics),  F14 (Mental and behavioural disorders due to use of cocaine),  F15 (Mental and behavioural disorders due to use of other stimulants),  F16 (Mental and behavioural disorders due to use of hallucinogens),  F18 (Mental and behavioural disorders due to use of volatile solvents),  F19 (Mental and behavioural disorders due to multiple drug use of other psychoactive substances)  T40 (Poisoning by narcotics and psychodysleptics),  T42 (Poisoning by antiepileptic, sedative-hypnotic and antiparkinsonism drugs),  X41 (Accidental poisoning by and exposure to antiepileptic, sedative-hypnotic, antiparkinsonism and psychotropic drugs, not elsewhere classified),  X42 (Accidental poisoning by and exposure to narcotics and psychodysleptics (hallucinogens), not elsewhere specified)  *ICD-9 codes:*  292 (Drug-induced psychoses),  304 (Drug dependence),  305X (Narcotic and medication misuse)  969 (Poisoning by psychotropic medications and narcotics),  *ICD-8 codes:*  304 (Drug dependence)  971 (Poisoning by narcotics) |
| Medications used in opioid use disorder treatment^c^ (from the Prescribed Drug Register) | *ATC-codes*:  N07BC01 (buprenorphine), N07BC02 (methadone), N07BC05 (levomethadone), and N07BC51 (buprenorphine, combinations) |
| Suspected criminal offences related to drug use (from the Register of Persons Suspected of Offences) | *Codes for suspected criminal offences:*  3070 (Driving under the influence of only drugs; *from the Law 1951:649, paragraphs 4 and 4a*),  5010 (Possession of drug only; *from the Narcotic Drug Act 1968:64*),  5011 (Use of drug only; *from the Narcotic Drugs Act 1968:64*),  5012 (Possession and use of drugs; *from the Narcotic Drugs Act 1968:64*) |

^a^ A combined outcome variable ‘any substance-related problems’ is created using all codes in the table.

^b^ The ICD-10 codes are used to construct the outcome variables as any substance-related problems, alcohol-related problems, and drug-related problems, while ICD-8, -9, and -10 codes are used for constructing study covariates as pre-existing substance-related problems, and maternal and paternal lifetime substance use-related problems.

^c^ The exact medications and the corresponding ATC-codes are selected for inclusion based on pharmacological treatment options reported in the Swedish Pharmaceutical guidelines (www.fass.se).

*Abbreviations*: ATC, Anatomical Therapeutic Chemical Classification System; ICD, International Classification of Diseases.

**Supplementary Table S2**. Swedish International Classification of Diseases (ICD) codes used to identify psychiatric disorders diagnosed before or on the index date^a^

| **Psychiatric disorders** | **Corresponding Swedish ICD-8 diagnostic codes** | **Corresponding Swedish ICD-9 diagnostic codes** | **Corresponding Swedish ICD-10 diagnostic codes** |
| --- | --- | --- | --- |
| **Neurodevelopmental disorders:** Autism spectrum disorders, attention-deficit/ hyperactivity disorder^b^, and Tourette syndrome and chronic tic disorder^c^ | 306.2 | 299, 307C, 314 | F84, F90, F95 |
| **Psychotic disorders:** Schizophrenia and other psychotic disorders | 295 (minus 295.5), 297, 298 (minus 298.09) | 295 (minus 295F), 297, 298 (minus 298A) | F20, F21, F22, F23, F24, F25 (minus F25.0), F28, F29 |
| **Bipolar disorders** | 296 (minus 296.00 and 296.20) | 296 (minus 296B) | F25.0, F30, F31, F34.0 |
| **Depressive disorders:** Major depressive disorder, persistent mood disorder, and unspecified mood disorder | 296.0, 296.2, 298.09 | 296B, 298A, 300E, 311 | F32, F33, F34 (minus F34.0), F38, F39 |
| **Anxiety disorders:** Phobic, anxiety, reaction to severe stress, and adjustment disorders | 300.0, 300.2, 307, 308.4 | 300A, 300C, 308, 309 | F40.0, F40.1, F40.2, F41.0, F41.1, F43 |
| **Eating disorders** | – | 307B, 307F | F50.0-F50.3, F50.9 |

^a^ Index date was defined as the date of the first hypochondriasis diagnosis among the exposed individuals and the corresponding date for their matched unexposed counterparts.

^b^ Individuals with attention-deficit/hyperactivity disorder (ADHD) were also identified by prescription of ADHD drugs, collected from the Prescription Drug Register, specifically Amphetamine (Anatomical Therapeutic Chemical [ATC] Classification System code: N06BA01), Dexamphetamine (N06BA02), Methylphenidate (N06BA04), Atomoxetine (N06BA09), and Lisdexamphetamine (N06BA12).

^c^ Tourette syndrome and chronic tic disorder were identified following the algorithm described elsewhere (Rück et al., 2015).

**Supplementary Table S3**. Hazard ratios (HRs) with 95% confidence intervals (CIs) for any substance-related problems, alcohol- and drug-related problems among females and males with a diagnosis of hypochondriasis, compared to matched unexposed individuals.

|  | **Individuals with hypochondriasis** | **Matched unexposed individuals** | **Model 1^a^** | **Model 2^b^** | **Model 3^c^** |
| --- | --- | --- | --- | --- | --- |
|  | **n (%)** | **n (%)** | **HR (95% CI)** | **HR (95% CI)** | **HR (95% CI)** |
| **Among females** |  |  |  |  |  |
| Included individuals | 2342 (100) | 23,420 (100) |  |  |  |
| Any substance-related problem | 216 (9.22) | 680 (2.90) | 3.47 (2.97-4.05) | 2.97 (2.52-3.49) | 2.90 (2.46-3.42) |
| Alcohol-related problem | 112 (4.78) | 438 (1.87) | 2.67 (2.17-3.29) | 2.28 (1.83-2.83) | 2.23 (1.79-2.77) |
| Drug-related problem | 154 (6.58) | 347 (1.48) | 4.81 (3.97-5.84) | 4.30 (3.49-5.29) | 4.15 (3.35-5.14) |
| **Among males** |  |  |  |  |  |
| Included individuals | 1787 (100) | 17,870 (100) |  |  |  |
| Any substance-related problem | 288 (16.12) | 1244 (6.96) | 2.57 (2.25-2.93) | 2.36 (2.05-2.70) | 2.33 (2.03-2.67) |
| Alcohol-related problem | 182 (10.18) | 702 (3.93) | 2.82 (2.39-3.32) | 2.57 (2.16-3.05) | 2.56 (2.15-3.04) |
| Drug-related problem | 174 (9.74) | 753 (4.21) | 2.47 (2.09-2.92) | 2.22 (1.86-2.65) | 2.17 (1.81-2.60) |

*Note*: All analyses in female and male participants were performed in the whole study cohort (i.e., including individuals with and without pre-existing substance-related problems).

^a^ Model 1 is adjusted for the matching variables (sex, birth year, and county of residence at the index date).

^b^ Model 2 is additionally adjusted for sociodemographic factors (place of birth, level of education, civil status, and household income).

^c^ Model 3 is additionally adjusted for maternal and paternal substance-related problems.

**Supplementary Table S4**. Distribution of sociodemographic, clinical, and parental characteristics in the sub-cohort of the Stockholm County residents with hypochondriasis diagnosed in specialist services or in primary care and their matched unexposed counterparts.

|  | **Individuals with hypochondriasis recorded in the NPR or VAL**  **n=3343^a^** | **Matched unexposed individuals**  **n=33,419** | **χ^2^ test or t-test** | **p-value** |
| --- | --- | --- | --- | --- |
|  | **n (%)** | **n (%)** |  |  |
| **Sex** |  |  | 0.0012 | 0.9722 |
| Female | 1949 (58.30) | 19,494 (58.33) |  |  |
| Male | 1394 (41.70) | 13,925 (41.67) |  |  |
| **Years of birth** |  |  | 0.0029 | 1.000 |
| ≤1939 | 49 (1.47) | 486 (1.45) |  |  |
| 1940-1959 | 323 (9.66) | 3230 (9.67) |  |  |
| 1960-1979 | 1125 (33.65) | 11249 (33.66) |  |  |
| 1980-1999 | 1762 (52.71) | 17614 (52.71) |  |  |
| ≥2000 | 84 (2.51) | 840 (2.51) |  |  |
| **Age at the index date^b^, years,**  **mean (SD)** |  |  |  |  |
| Among all individuals | 36.84 (13.67) | 36.84 (13.65) | -0.01 | 0.9917 |
| Among females | 36.72 (13.68) | 36.72 (13.66) | 0 | 0.9966 |
| Among males | 37.02 (13.66) | 37.01 (13.64) | -0.02 | 0.9836 |
| **Place of birth** |  |  | 123.80 | <0.0001 |
| Sweden | 2731 (81.69) | 24328 (72.80) |  |  |
| Abroad | 612 (18.31) | 9091 (27.20) |  |  |
| **Educational level** |  |  | 40.94 | <0.0001 |
| Elementary education | 466 (13.94) | 4727 (14.14) |  |  |
| Secondary education | 1165 (34.85) | 12024 (35.98) |  |  |
| Higher education | 1684 (50.37) | 15850 (47.43) |  |  |
| Unknown/missing | 28 (0.84) | 818 (2.45) |  |  |
| **Civil status** |  |  | 8.42 | 0.148 |
| Single, divorced or widowed | 2308 (69.04) | 22246 (66.57) |  |  |
| Married or cohabiting | 1026 (30.69) | 11083 (33.16) |  |  |
| Unknown/missing | 9 (0.27) | 90 (0.27) |  |  |
| **Disposable household income level** |  |  | 33.44 | <0.0001 |
| Lowest 20% | 792 (23.69) | 7005 (20.96) |  |  |
| Middle 60% | 1933 (57.82) | 19535 (58.45) |  |  |
| Top 20% | 594 (17.77) | 6278 (18.79) |  |  |
| Unknown/missing | 24 (0.72) | 601 (1.80) |  |  |
| **Maternal substance-related problems** |  |  | 100.4075 | <.0001 |
| No | 2603 (77.86) | 24070 (72.02) |  |  |
| Yes | 191 (5.71) | 1412 (4.23) |  |  |
| Unknown/missing | 549 (16.42) | 7937 (23.75) |  |  |
| **Paternal substance-related problems** |  |  | 94.4854 | <.0001 |
| No | 2424 (72.51) | 22366 (66.93) |  |  |
| Yes | 370 (11.07) | 3116 (9.32) |  |  |
| Unknown/missing | 549 (16.42) | 7937 (23.75) |  |  |
| **Pre-existing alcohol-related problems** |  |  | 12.26 | 0.0005 |
| No | 3122 (93.39) | 31,686 (94.81) |  |  |
| Yes | 221 (6.61) | 1733 (5.19) |  |  |
| **Pre-existing drug-related problems** |  |  | 10.03 | 0.0015 |
| No | 3137 (93.84) | 31,779 (95.09) |  |  |
| Yes | 206 (6.16) | 1640 (4.91) |  |  |
| **History of psychiatric disorders** |  |  |  |  |
| Any psychiatric disorders | 1732 (51.81) | 4407 (13.19) | 3258.87 | <0.0001 |
| Neurodevelopmental disorders | 213 (6.37) | 1132 (3.39) | 76.78 | <0.0001 |
| Psychotic disorders | 88 (2.63) | 329 (0.98) | 73.59 | <0.0001 |
| Bipolar disorders | 96 (2.87) | 382 (1.14) | 70.76 | <0.0001 |
| Depressive disorders | 827 (24.74) | 2172 (6.50) | 1349.31 | <0.0001 |
| Anxiety-related disorders | 1367 (40.89) | 2201 (6.59) | 4081.01 | <0.0001 |
| Eating disorders | 149 (4.46) | 514 (1.54) | 146.22 | <0.0001 |
| **Age at the first recorded substance-related problem, years, mean (SD)** |  |  |  |  |
| Among all individuals | 40.12 (14.80) | 37.94 (14.92) | -2.06 | 0.0396 |
| Among females | 39.31 (13.55) | 37.73 (14.80) | -1.20 | 0.2296 |
| Among males | 41.44 (16.65) | 38.31 (15.13) | -1.76 | 0.0784 |
| **Duration of follow up, years, mean (SD)** |  |  |  |  |
| For all individuals | 4.70 (4.47) | 4.85 (4.59) | 1.81 | 0.0707 |
| For females | 4.72 (4.44) | 4.91 (4.53) | 1.52 | 0.1276 |
| For males | 4.69 (4.50) | 4.81 (4.63) | 1.09 | 0.2778 |

*Note*. Statistical comparisons were conducted using Chi-square tests for categorical variables and t-tests for continuous variables; p-values from the corresponding tests are provided in the last column.

^a^ The total number of individuals with hypochondriasis (n=3343) includes 1235 (36.94%) with diagnoses recorded only in primary care (identified in the VAL), 1753 (52.44%) with diagnoses recorded only in specialist care (identified in the NPR), and 355 (10.62%) with diagnoses recorded in both sources.

^b^ Index date refers to the date of the first diagnosis of hypochondriasis among the exposed individuals and the corresponding date among their matched unexposed counterparts

*Abbreviations*: NPR, the National Patient Register; SD, standard deviation; VAL, the Swedish acronym for the Region Stockholm’s healthcare administration's database.

**Supplementary Table S5**. Hazard ratios (HRs) with 95% confidence intervals (CIs) for any substance-related problems and, specifically, for alcohol- and drug-related problems, among individuals with a diagnosis of hypochondriasis assigned in specialist services or in primary care in the Stockholm County only, compared to matched unexposed individuals, further adjusted for different groups of lifetime psychiatric comorbidities

|  | **Initial Model 2^a^ additionally adjusted for the following disorder groups (one group at a time)** | | | | | |
| --- | --- | --- | --- | --- | --- | --- |
|  | **Neurodevelopmental disorders** | **Psychotic disorders** | **Bipolar disorders** | **Depressive disorders** | **Anxiety disorders** | **Eating disorders** |
|  | **HR (95% CI)** | **HR (95% CI)** | **HR (95% CI)** | **HR (95% CI)** | **HR (95% CI)** | **HR (95% CI)** |
| **Sub-cohort of individuals residing in Region Stockholm in 1997-2020** |  |  |  |  |  |  |
| Any substance-related problem | 1.54 (1.33-1.78) | 1.54 (1.33-1.79) | 1.54 (1.33-1.79) | 1.16 (0.99-1.36) | 1.08 (0.91-1.28) | 1.58 (1.37-1.83) |
| Alcohol-related problem | 1.60 (1.33-1.93) | 1.63 (1.36-1.97) | 1.61 (1.34-1.94) | 1.18 (0.97-1.44) | 1.15 (0.93-1.41) | 1.65 (1.37-1.98) |
| Drug-related problem | 1.61 (1.33-1.96) | 1.61 (1.32-1.96) | 1.64 (1.35-2.00) | 1.29 (1.05-1.59) | 1.17 (0.94-1.46) | 1.67 (1.38-2.03) |
| **Without pre-existing substance-related problems^b^** |  |  |  |  |  |  |
| Any substance-related problem | 1.62 (1.32-1.99) | 1.64 (1.33-2.01) | 1.61 (1.31-1.98) | 1.35 (1.09-1.69) | 1.36 (1.07-1.73) | 1.63 (1.32-2.01) |
| Alcohol-related problem | 1.48 (1.13-1.95) | 1.52 (1.16-1.99) | 1.48 (1.12-1.94) | 1.24 (0.92-1.66) | 1.28 (0.94-1.75) | 1.50 (1.15-1.98) |
| Drug-related problem | 1.96 (1.48-2.59) | 1.94 (1.46-2.57) | 1.97 (1.49-2.60) | 1.59 (1.18-2.15) | 1.59 (1.15-2.20) | 1.98 (1.50-2.62) |

*Note*: The analysis is based on a subset of individuals from the study population who were living in Stockholm County between 1997-2020. Among them, individuals with the first hypochondriasis diagnosis assigned in specialist services (according to the National Patient Register) or in primary care (according to the Region Stockholm’s healthcare administration's database) were identified. They were then matched to up to 10 unexposed individuals from the general population of Stockholm County who had no hypochondriasis diagnosis before and at the date when the exposed person was diagnosed.

^a^ Model 2 is adjusted for the matching variables (sex, birth year, and county of residence at the index date) and additionally adjusted for sociodemographic factors (place of birth, level of education, civil status, and household income).

^b^ The sub-cohort is restricted to individuals with neither pre-existing alcohol-related problems nor drug-related problems

**Supplementary Table S6**. Hazard ratios (HRs) with 95% confidence intervals (CIs) for any substance-related problems, alcohol- and drug-related problems among individuals with hypochondriasis, compared to matched unexposed individuals, using the new outcome definition (sensitivity analysis #1) and in a sub-cohort restricted to individuals with complete data on all covariates (sensitivity analysis #2)

|  | **Individuals with hypochondriasis** | **Matched unexposed individuals** | **Model 1^a^** | **Model 2^b^** | **Model 3^c^** |
| --- | --- | --- | --- | --- | --- |
|  | **n (%)** | **n (%)** | **HR (95% CI)** | **HR (95% CI)** | **HR (95% CI)** |
| **Without medication for opioid use disorders in the outcome definition** |  |  |  |  |  |
| Included individuals | 4129 (100) | 41,290 (100) |  |  |  |
| Any substance-related problem | 501 (12.13) | 1906 (4.62) | 2.89 (2.62-3.20) | 2.59 (2.33-2.88) | 2.55 (2.30-2.84) |
| Alcohol-related problem | 294 (7.12) | 1140 (2.76) | 2.76 (2.42-3.14) | 2.46 (2.15-2.81) | 2.43 (2.12-2.78) |
| Drug-related problem | 325 (7.87) | 1082 (2.62) | 3.22 (2.84-3.65) | 2.88 (2.52-3.30) | 2.82 (2.46-3.24) |
| **Among individuals with complete information on all covariates** |  |  |  |  |  |
| Included individuals | 3510 (100) | 32,283 (100) |  |  |  |
| Any substance-related problem | 448 (12.76) | 1616 (5.01) | 2.92 (2.62-3.25) | 2.58 (2.30-2.89) | 2.56 (2.28-2.87) |
| Alcohol-related problem | 272 (7.75) | 969 (3.00) | 2.94 (2.56-3.38) | 2.60 (2.25-3.01) | 2.58 (2.23-2.99) |
| Drug-related problem | 285 (8.12) | 919 (2.85) | 3.09 (2.70-3.55) | 2.73 (2.35-3.16) | 2.71 (2.33-3.15) |

^a^ Model 1 is adjusted for the matching variables (sex, birth year, and county of residence at the index date).

^b^ Model 2 is additionally adjusted for sociodemographic factors (place of birth, level of education, civil status, and household income).

^c^ Model 3 is additionally adjusted for maternal and paternal substance-related problems.
